# Common miRNAs, candidate genes and their interaction network across four subtypes of epithelial ovarian cancer

**DOI:** 10.6026/97320630017748

**Published:** 2021-08-31

**Authors:** Rinki Singh, Anup Som

**Affiliations:** 1 Centre of Bioinformatics, Institute of Interdisciplinary Studies, University of Allahabad, Prayagraj - 211002, India

**Keywords:** Epithelial ovarian cancer, Differential gene expression, Biomarkers, Gene ontology, Survival analysis, miRNA-mRNA network

## Abstract

Epithelial ovarian cancer (EOC) is categorized into four major histological subtypes such as clear cell carcinoma (CCC), endometrioid carcinoma (EC), mucinous carcinoma (MC), and serous carcinoma (SC). Heterogeneity of the EOC leads to different clinical outcomes of the disease, although all the subtypes are originated from the same layer of tissue. Therefore, it is of interest to identify the common candidate genes, miRNA and their interaction network in four the subtypes of EOC. A comparative gene expression analysis identified 248 common differentially expressed genes (DEGs) in the four subtypes of EOC. Identified common DEGs were found to be enriched in cancer specific pathways. A protein-protein interaction (PPI) network of the common DEGs were constructed, and subsequent module and survival analyses identified seven key candidate genes (CCNB1, CENPM, CEP55, RACGAP1, TPX2, UBE2C, and ZWINT). We also documented 10 key candidate miRNAs (hsa-mir-16-5p, hsa-mir-23b-3p, hsa-mir-34a-5p, hsa-mir-103a-3p, hsa-mir-107, hsa-mir-124-3p, hsa-mir-129-2-3p, hsa-mir-147a, hsa-mir-205-5p, and hsa-mir-195-5p) linked to the candidate genes. These derived data find application in the understanding of EOC.

## Background:

Ovarian cancer is the eighth most common carcinoma that is associated with a high mortality rate among women worldwide [1]. Ovarian cancer is mostly detected at the advanced stage because of its asymptomatic growth and lack of reliable screening techniques. It was reported that up to 90% of all ovarian cancer have an epithelial origin [2]. Epithelial ovarian cancer (EOC) is further categorized into four histological subtypes specifically clear cell carcinoma, endometrioid, mucinous, and serous carcinomas. Each of these subtypes is associated with different genetic factors, molecular events, and mRNA expression profiles that make each subtype a distinct disease, although each subtype have epithelial origin [3]. Protein CA125 (cancer antigen 125) is a well-known serum biomarker used for the detection of ovarian cancer. However, its expression level is not uniform across all. Thus, CA125 is not a highly specific diagnostic marker for ovarian cancer. Patients with different subtypes respond in a different way to the same treatment and also have different prognoses due to its heterogeneity, which complicates ovarian cancer treatment. Therefore, identifying new reliable biomarkers common in subtypes of EOC are required for the improved treatment of EOC. Microarray experiments are producing massive quantities of gene expression and other functional genomic data, which give us a deeper systems-level insight of a disease. Protein-protein interaction (PPI) network analysis is a powerful approach for finding the candidate genes because it is used to represent, compute and model intracellular interactions to gain insights into the molecular architecture of any complex process [4-5]. Substantial efforts have been made to discover diagnostic and prognostic biomarkers of serous subtype using gene expression data [6-7]. However, a limited number of studies were conducted on the comparative gene expression analysis of the subtypes of EOC. One such study performed by Madore et al. defines the molecular characteristics of serous and endometrioid carcinomas to address the problems with the current histopathological classification methods [8]. Another study, Pamula-Pilat et al. analyzed transcriptomic profiles of three histological subtypes specifically clear cell carcinoma, endometrioma and serous carcinomas to identify molecular differences that explained the different responses of these subtypes against chemotherapy [9]. In our recent work, we identified 13 hub genes that were functionally interacted among the four major subtypes [10]. Therefore, it is of interest to identify and document the common miRNAs, candidate genes and their interaction network across the four subtypes of EOC.

## Materials & Methods:

### Data retrieval:

**Figure 1:**
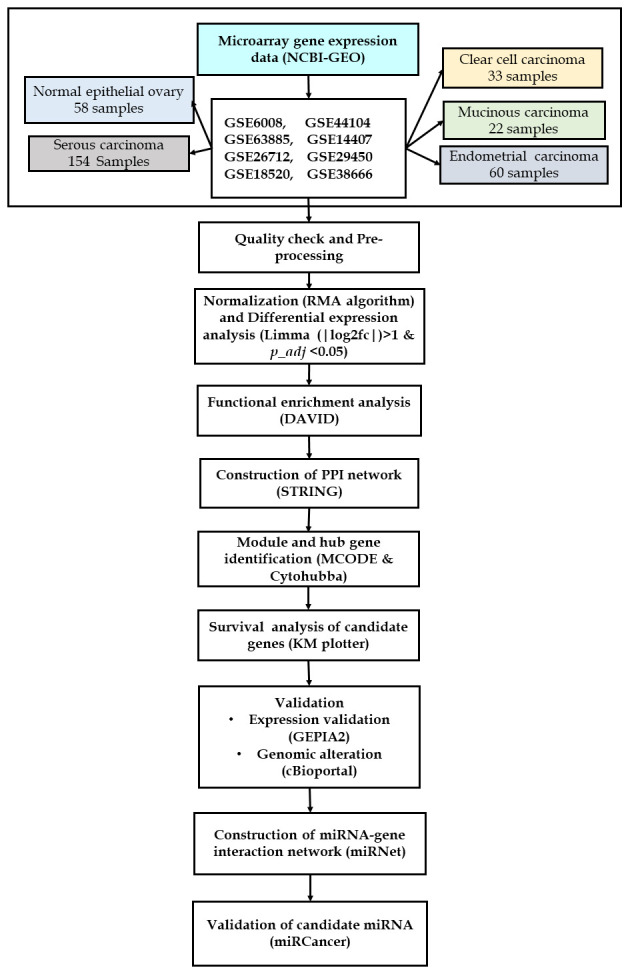
Flowchart of the methodology implemented in this study.

This study includes 333 microarray samples from eight gene expression datasets (GSE6008, GSE44104, GSE63885, GSE14407, GSE26712, GSE29450, GSE18520, and GSE38666) that belongs to two Affymetrix platforms namely GPL750 and GPL96. All the datasets were retrieved from Gene Expression Omnibus (https://www.ncbi.nlm.nih.gov/). Of the total 333 samples, 39 samples belong to clear cell carcinoma, 60 belong to endometrioma, 22 belong to mucinous carcinoma, 154 belong to serous carcinoma, and 58 belong to normal samples (Table 1 - see PDF). A flowchart of this study is given in Figure 1.

### Data processing:

Raw microarray data contains different sources of noise such as due to the experimental handling, efficacy, and efficiency of the probe, noisy signal due to labelling efficiency, between slide variation and other factors. Hence, it is necessary to pre-process the raw data before the expression measures that can be used for further analysis. The raw data were standardized and transformed into expression value using the affy package of Bioconductor. Pre-processing of the raw data from the same platform was done together. The robust multi-array average (RMA) algorithm was used for pre-processing of the microarray data that included background signal correction, data normalization, and probe summarization. For the batch correction process limma package was used.

### Differential expression analysis:

Limma (Linear models for microarray data) package was used for the identification of DEGs in different subtypes of EOC by comparing it with the normal ovarian surface epithelium samples. Further, the false-discovery rate arising due to multiple hypothesis testing was minimized through Benjamini-Hochberg's method. We considered absolute logarithmic fold change (|log2fc|)>1 and adjusted p-value (adj) <0.05 for the identification of DEGs. We used Venn diagram (http://bioinformatics.psb.ugent.be/webtools/Venn/) to identify overlapped/common DEGs among four subtypes of EOC.

### Functional enrichment analysis of DEGs:

Gene ontology (GO) and KEGG pathway enrichment analysis of the common DEGs in four subtypes of EOC were conducted using the DAVID online tool (Sherman al., 2007). GO terms consist of three categories that included biological process (BP), molecular function (MF), and cellular component (CC). Significance testing of the matched terms was performed and a p-value < 0.05 was considered to select the significantly enriched terms.

### Construction of PPI network and module analysis:

A PPI network of the identified common DEGs was constructed by the STRING database (https://string-db.org/) with an interaction score > 0.7. Subsequently, potential module from the PPI network was identified by the Cytoscape plugin Molecular Complex Detection (MCODE). Further, based on the highest degree of connectivity, the top 10 genes were identified. We also performed GO analysis to gain insights into the biological functions of the genes in the largest module.

### Survival analysis and validation of module genes:

Progression-free survival (PFS) analysis of the genes in the largest module was performed using Kaplan-Meier plotter online tool (http://kmplot.com/analysis/) to assess the effect of genes on the progression-free survival of ovarian cancer patients. We considered the criteria of log-rank P-value<0.05 and hazard ratio (HR) with a confidence interval 95% for the identification of significant genes. Genes that were significantly associated with the progression-free survival of the EOC patients were referred to as candidate genes.

Next, to investigate the genetic alteration information of these significant genes, we used cBioCancer Genomics Portal (https://www.cbioportal.org/). Further, the expression level of these significant genes was verified by GEPIA2 web-based tool using a threshold of |logfc|>1 and padj<0.01 (http://gepia2.cancer-pku.cn/#index).

### miRNA–candidate gene network:

MicroRNAs (miRNAs) can regulate nearly all-biological processes and have demonstrated that their dysregulation is implicated with human cancer. Thus, in addition to the candidate genes, we also aim to find out the regulatory miRNAs that target all the candidate genes. It is well known that a single miRNA can target many genes and also multiple miRNAs can regulate a single gene. The miRNet database (https://www.mirnet.ca/) is used for the identification of candidate genes targeting miRNAs and the construction of miRNA-candidate gene interaction network (i.e., the interactions of miRNAs with their target genes are called miRNA-candidate gene network).

## Results:

### Identification of DEGs in the four subtypes of EOC:

**Figure 2:**
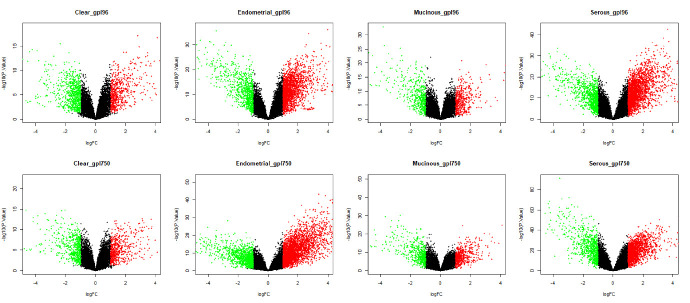
Volcano plots represent the proportion of genes found to be differentially expressed in each subtype of EOC. The X-axis represents the log2 transformed of fold change ratios and Y-axis is the log10 transformed adjusted p-value. Green dots: down regulated DEGs, Red dots: up regulated DEGs based on |logfc|>1 and p<0.05.

**Figure 3:**
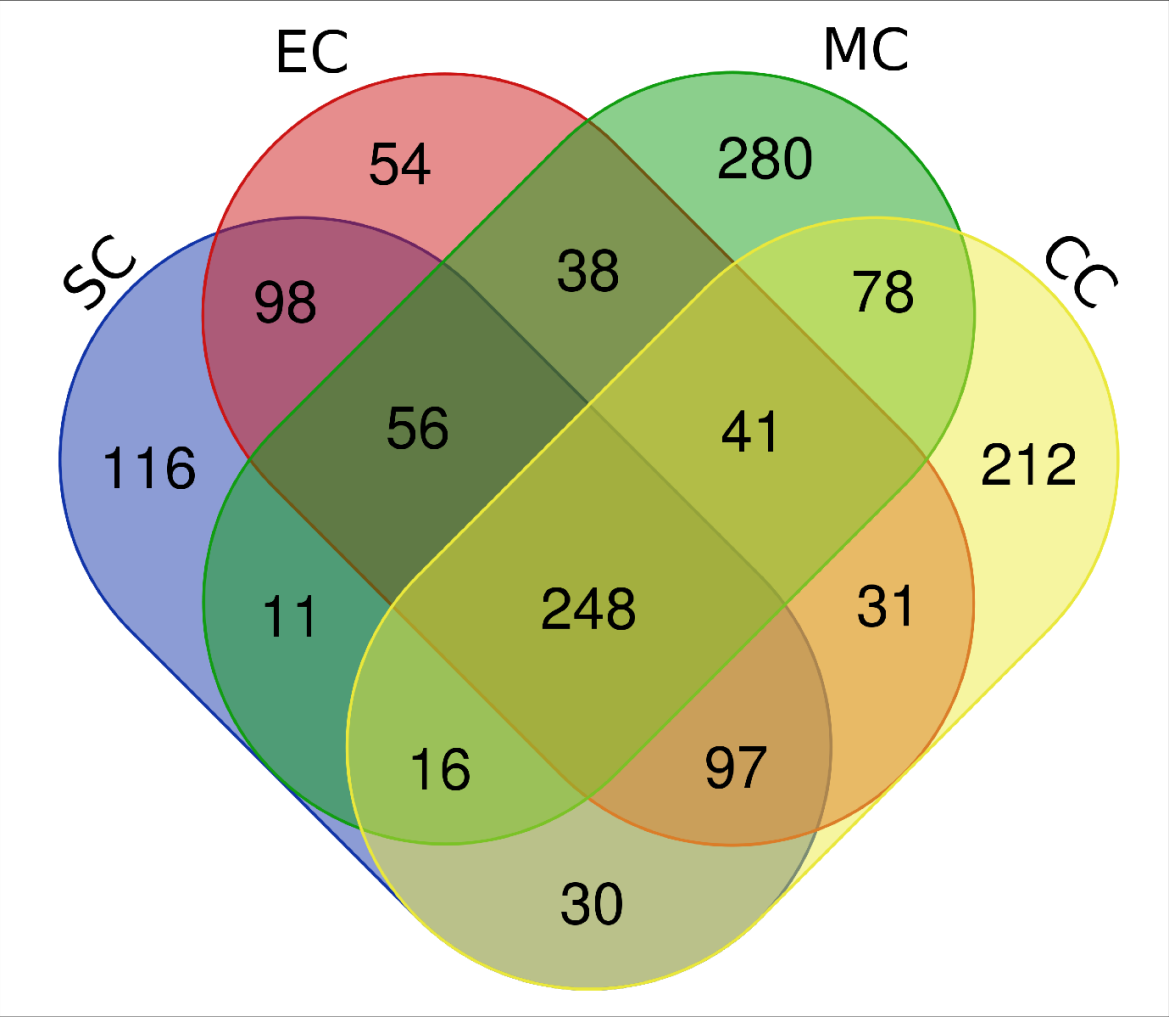
Venn diagram shows 248 common DEGs among serous carcinoma (SC), endometrioid carcinoma (EC), mucinous carcinoma (MC), and clear cell carcinoma (CCC).

Differential gene expression analysis was carried out to find the genes that were differentially expressed between cancerous and normal conditions. Differentially expressed genes (DEGs) were identified based on the criteria of i) absolute logarithmic fold change (|log2fc|)>1 and ii) adjusted p-value (padj)<0.05. The proportion of genes that were differentially expressed in each subtype is represented using the volcano plot (Figure 2). Further, differential gene expression analysis revealed a total of 753 genes (478 genes upregulated and 275 genes downregulated) were differentially expressed in clear cell carcinoma. Similarly, 663 genes (405 upregulated and 266 downregulated) in endometrioid, 768 genes (upregulated 480 and downregulated 278) in mucinous, and 672 genes (upregulated 427 and downregulated 245) in serous carcinoma were differentially expressed. Finally, we identified a total of 248 DEGs (upregulated 128 and downregulated 120) that are common in all four subtypes of EOC. These 248 genes are called "common DEGs". In Figure 3, a Venn diagram shows common/overlapped DEGs among the four subtypes of EOC.

### Function and pathway enrichment analysis of DEGs:

GO and pathway enrichment analyses were conducted to identify the biological significance of common DEGs. Results of GO analysis exhibited that extracellular matrix organization, angiogenesis, membrane organization, liver development, cellular response to vascular endothelial growth factor stimulus were the top five significant biological processes of the common DEGs (Figure 4A - see PDF). Similarly, heparin binding, cadherin binding involved in cell-cell adhesion, protein kinase, protein binding and protein domain specific binding were the top five significant molecular functions, and extracellular exome, extracellular matrix, extracellular space, and proteinaceous extracellular matrix were the top five significant cellular component terms (Figure 4B&4C - see PDF). Pathway enrichment analysis identified biosynthesis of amino acids, glycolysis/gluconeogenesis, oocyte meiosis, phagosome, cell cycle, tyrosine metabolism, RAP1 signaling pathway, drug metabolism-cytochrome P450, carbon metabolism, PI3K-AKT signaling pathway, and pathways in cancer are common pathways that were shared among the four subtypes of EOC (Table 2 - see PDF).

### PPI network construction and module identification:

STRING database was used for the identification of PPIs among the common DEGs and retrieved a PPI network of 137 nodes (genes) and 264 edges (interactions) at the confidence score > 0.7 and P-value <1.0e-16 (PPI enrichment). The PPI network was exported in the Cytoscape and island nodes were removed. Finally, the PPI network contained 103 nodes with 238 interactions (Figure 5A - see PDF). Further, significant module was identified for a deeper understanding of the cellular organization, processes, and functions via the MCODE plugin. Only one module (Module 1: nodes = 15, edges=101, MCODE score = 14.429) was identified based on the criteria that a module should has at least 5 nodes and network density ≥0.50. Besides module analysis, we carried out hub gene analysis and identified the top 10 genes with the highest degree (i.e., top 10 hub genes) belonged to Module 1. Thus, we considered Module 1 (the largest module) for further downstream analysis (Figure 5B - see PDF). Additionally, GO enrichment analysis was carried out to gain insights into the distinct functions of the genes in the largest module. It was found that genes in the Module 1 were mostly involved in cell cycle processes like mitotic spindle midzone assembly, mitotic spindle elongation and positive regulation of ubiquitin-protein ligase activity. 

### Survival analysis, genetic alteration and expression validation of candidate genes:

**Figure 6:**
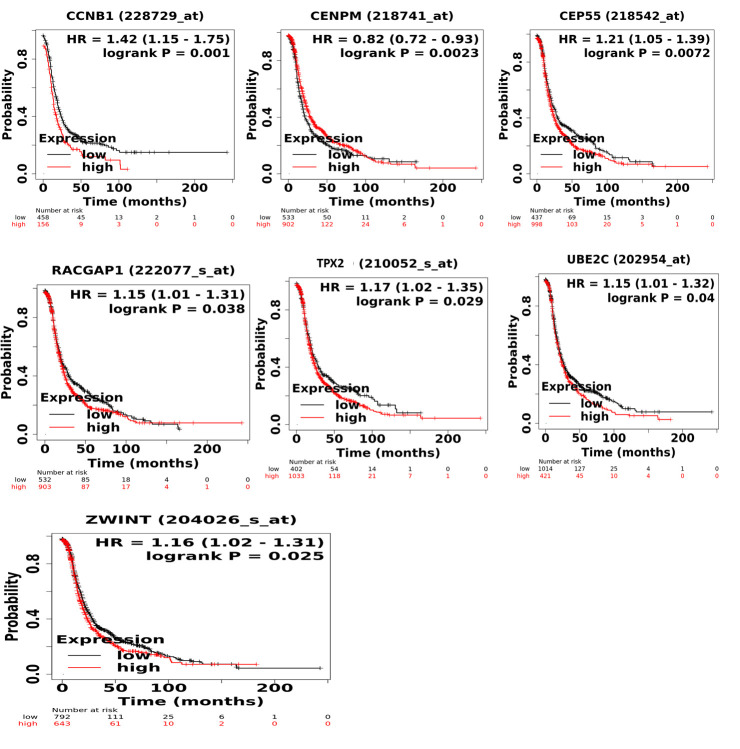
Progression free survival (PFS) analysis of module genes in EOC patients. Survival curves are based on the low and high expression of the module genes in EOC patients. Log-rank P < 0.05 was considered statistically significant.

**Figure 7:**
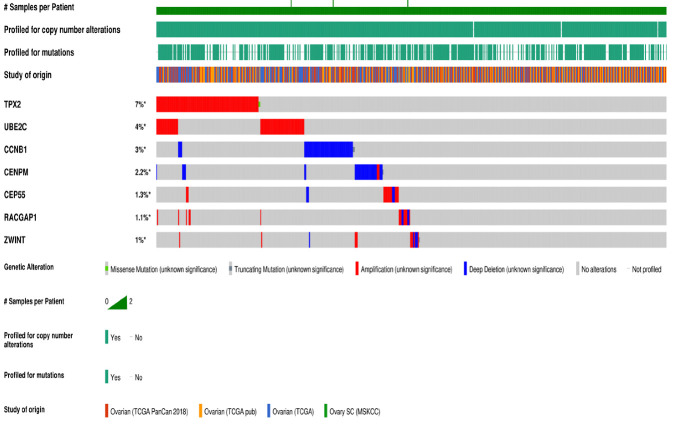
The genetic alterations occur in candidate genes are shown through a visual summary across a set of ovarian serous cystadenocarcinoma samples (data from TCGA, PanCancer Atlas).

**Figure 8:**
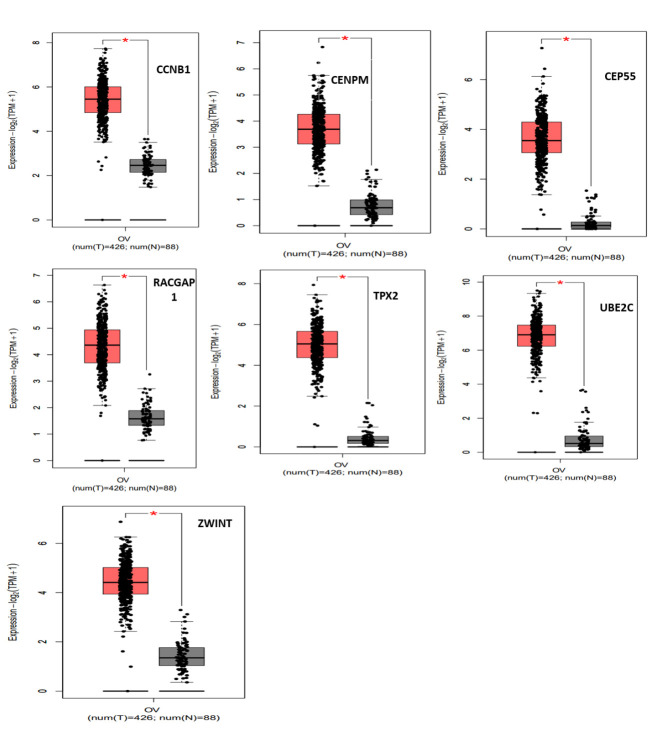
Expression level of the candidate genes in EOC tissues and normal tissues.

The prognostic information of the genes (15 genes) in the largest module was retrieved by the Kaplan-Meier plotter database. A total seven genes were found to be significantly associated with the PFS of EOC patients. It has been identified that decreased expression of CCNB1, CEP55, RACGAP1, TPX2, UBE2C, and ZWINT and high expression of CENPM associated with better PFS in EOC patients as shown in Figure 6. Subsequently, the genetic alteration information of the seven candidate genes was evaluated by using cBioPortal (https://www.cbioportal.org/) as illustrated in Figure 7. TPX2 was the most frequently altered gene (7 %) as compared to other candidate genes. Further analysis found that among all types of mutations such as amplification, deep deletion, fusion, inframe mutation, missense mutation, and truncating mutation, the percentage of amplification is the highest. Furthermore, the expression validation of the candidate genes shows that their expression levels are high in EOC as compared to normal tissues shows in Figure 8. Results of expression analysis obtained from the GEPIA2 database are in accordance with the analyzed GEO datasets, which validate that the expression level of the candidate genes is high in tumor tissues.

### miRNA–candidate gene network:

**Figure 9:**
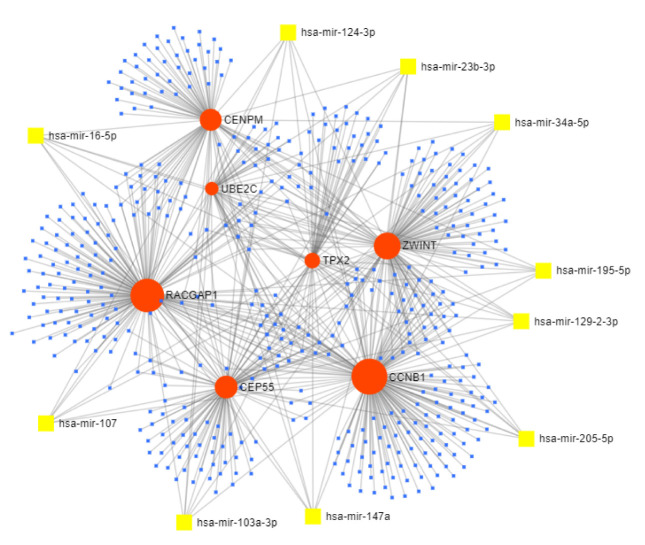
miRNA-candidate gene interaction network. Candidate genes are presented in red circles and miRNAs are shown in blue and Yellow Square. Yellow squares represented the miRNAs that target all the candidate genes.

Construction of miRNA-candidate gene network identified an interaction network consisting of seven candidate genes and 477 miRNAs with 731 interactions (Figure 9). Based on the degree of connectivity, CCNB1 (degree =169) is the top most candidate gene targeted by 169 miRNAs followed by RACGAP1 (degree =153), ZWINT (degree = 122), and CEP55 (degree = 101), CENPM (degree = 87), TPX2 (degree = 55), UBE2C (degree = 44). Further, we identified 10 miRNAs (namely hsa-mir-16-5p, hsa-mir-23b-3p, hsa-mir-34a-5p, hsa-mir-103a-3p, hsa-mir-107, hsa-mir-124-3p, hsa-mir-129-2-3p, hsa-mir-147a, hsa-mir-195-5p, and hsa-mir-205-5p) that target all the candidate genes (called as candidate miRNAs) (Table 3 - see PDF) and validation of these candidate miRNAs was performed using the miRCancer database (http://mircancer.ecu.edu/) that demonstrated the role of miRNAs in EOC progression.

## Discussion:

Epithelial ovarian cancer is one of the most lethal gynecological cancers worldwide due to its heterogeneity, delayed diagnosis as well as recurrence and drug resistance. In this study, we identified 248 DEGs that are common across all four types of EOC using comparative gene expression and integrated bioinformatics analyses. It is well known that cancer is a complex and heterogeneous disease, which is characterized, by extensive genomic abnormalities and aberrations in gene expression that causes dysregulation of various signaling pathways. Gene ontology and pathway enrichment analysis provides substantial support that each subtype of EOC follows common molecular mechanism in the tumor progression. Biological molecules usually exert their functions through a complex interplay of interactions. Construction of PPI network among the common DEGs and module analysis identified the largest cluster of 15 genes that formed a close circuitry in the PPI network. Subsequently, survival analysis of module genes demonstrated that seven genes out of 15 were significantly associated with PFS of EOC patients. These significant genes (CCNB1, CENPM, CEP55, RACGAP1, TPX2, UBE2C, and ZWINT) might have diagnostic, prognostic, and therapeutic applications in EOC patients irrespective of their subtypes hence, referred as candidate genes. Genetic alteration information of the candidate genes illustrated that frequent mutation in these genes associated with ovarian cancer. Furthermore, the result of expression analysis validates that candidate genes are upregulated in EOC. Besides the two-tier validation of the candidate genes, we did a comprehensive literature search on the candidate genes and found that the candidate genes are involved in various events of the cell cycle such as microtubule organization, kinetochore complex formation, regulation at G2/M transition, ubiquitination process, etc. Cell cycle is one of the most fundamental and highly controlled processes that take place in cell. The dysregulation of the cell cycle is a hallmark of cancer development. A concise description of the candidate genes pertaining to cancer is described further.

CCNB1 (G2/Mitotic-specific cyclin B1) is a regulatory protein that regulates the cell cycle process at the phase of G2/M transition. It has been reported that CCNB1 shows increased expression levels throughout the cell cycle in considerable cancers including ovarian carcinoma [19]. CEP55 and TPX2 are essential for normal mitotic spindle function during cell division. These genes play key role in microtubule assembly by interacting directly or indirectly with various other proteins like microtubule-binding proteins, motors and nucleation factors. Growing evidence demonstrates that overexpression of CEP55 and TPX2 have been implicated in the development of the ovarian and numerous other carcinomas [20- 22]. CENPM and ZWINT are participated in kinetochore complex formation that have central role in the assembly of kinetochore proteins, chromosome alignment kinetochore-microtubule attachment, spindle assembly checkpoint function, and ensures that chromosomes are divided equally between daughter cells which is required for successful mitotic division. Lately, overexpression of CENPM and ZWINT has been reported in human malignancies [23-24]. RACGAP1 (Rac GTPase-activating protein 1), a central spindle complex plays a very important role in controlling various cellular processes including invasive migration and metastasis through the binding of activated form of RhoGTPase. Previous studies reported the overexpression of RACGAP1 lead to gastric, ovarian, colorectal, and several other cancers, implying its role in promoting tumor progression [25-27]. A number of studies showed high expression of UBE2C is associated with aggressive progression and poor outcomes of various types of cancer [27-30]. miRNAs are small, endogenous, non-coding RNAs with a length of 19-25 nucleotides that participate in numerous biological processes such as cell proliferation, invasion and migration by regulating the expression of target genes at the post-transcriptional level [31]. Dysregulation of miRNAs plays crucial role in tumor initiation and progression by acting as either tumor suppressors or oncogene [32]. Several studies have shown that large numbers of miRNAs are dysregulated in EOC [33-35]. A study by Yang et al (2019) reported that miRNA-802 is involved in the ovarian cancer development process by regulating the expression level of the YWHAZ gene [36]. Another study by Zhang et al (2019) found that miRNA-574-3p inhibits ovarian cancer progression through expression regulation of EGFR [37].

UBE2C (Ubiquitin-conjugating enzyme E2C) involved in the modification of abnormal or short-lived proteins by the addition of ubiquitin and lead them toward degradation, a of miRNA-21 negatively correlated with the PTEN target gene, and suppression of miRNA-21 inhibits ovarian cancer progression [38]. Thus, these findings suggested miRNA as a diagnostic, therapeutic, and prognostic biomarker of EOC. Thus, miRNA and candidate gene interaction networks were constructed to understand the regulatory mechanism of these genes (Figure 9). This work reported 10 miRNAs that regulate the expression of the candidate genes. It was reported that downregulation of hsa-mir-16-5p, has-mir-34a, has-mir-107, and has-mir-124 are associated with ovarian cancer [39-41], whereas, upregulation of hsa-mir-205-5p drives cell proliferation and metastasis in ovarian carcinoma [42]. Though it was reported that hsa-mir-103a-3p, hsa-mir-23b-3p, hsa-mir-129-2-3p, hsa-mir-147a, and hsa-miR-195-5p contribute to tumor growth [43-46], but their exact role in ovarian cancer needs to be examined.

## Conclusion:

Determination of underlying molecular interaction networks involved in the formation and progression of the four subtypes of EOC may aid in the treatment of the disease. A protein-protein interaction (PPI) network analysis of the common DEGs helped to glean seven key candidate genes (CCNB1, CENPM, CEP55, RACGAP1, TPX2, UBE2C, and ZWINT). We also reported 10 key candidate miRNAs (hsa-mir-16-5p, hsa-mir-23b-3p, hsa-mir-34a-5p, hsa-mir-103a-3p, hsa-mir-107, hsa-mir-124-3p, hsa-mir-129-2-3p, hsa-mir-147a, hsa-mir-205-5p, and hsa-mir-195-5p) linked to the candidate genes. These derived data find application in the understanding of EOC.
